# Exploring and understanding different perspectives on the experience of engaging with death doulas and those in activity-aligned roles toward the end of life: An integrative review

**DOI:** 10.1177/26323524251389218

**Published:** 2025-11-14

**Authors:** Samara Gordon Wexler, Catherine Walshe

**Affiliations:** 1 International Observatory on End of Life Care, Lancaster University, UK; 2 Watson Foundation, New York, NY, USA

**Keywords:** doulas, death, terminal care, palliative care, experiences

## Abstract

**Background:**

The death doula movement is expanding due to dissatisfaction with the medicalization of death and dying. Existing reviews focus on exploring and defining the death doula’s role in providing care. However, the experiences of death doulas or those performing aligned activities for the dying person, families, and healthcare professionals have not been synthesized.

**Objective:**

To explore the experiences of engaging with death doulas and those performing aligned activities from multiple perspectives (including the dying person, their families, health and social care professionals, and death doulas or those in activity-aligned roles themselves).

**Design:**

A systematically constructed integrative review.

**Data sources and methods:**

Medline, CINAHL, Scopus, and Lens.org (searched September 2024) for concepts related to death doula and palliative care. Inclusion criteria: discussion of death doula or aligned activities; dying persons, families, doulas, or healthcare workers’ experiences with death doulas; any study type; any year; in English. Exclusion criteria: birthing, labor, or maternal doulas/midwives. Non-human death, life-limiting illnesses in people who are not in the end-of-life phase, or healthcare professionals or social workers, reviews, protocols, and abstracts. Papers were coded iteratively and synthesized into final themes. Quality appraisal was done using Mixed Method Appraisal Tool scoring.

**Results:**

Papers (*n* = 33) from six countries. Careful analysis and synthesis resulted in the creation of six themes: emotions before and after the engagement, transforming fear through knowledge and literacy, objective companionship, the death doula as a mediator, the death doula “cycle,” and the tension between flexibility and regulation.

**Conclusion:**

The limited evidence from literature, including experiential perspectives outside of reports from death doulas or those in aligned-activities roles, indicates that research should continue to explore the benefits of adding these roles to end-of-life care. Positive experiences of engaging a doula or with those performing aligned activities appear related to role flexibility, which seems to facilitate other favorable experiences. However, flexibility also seems to be a cause of role confusion and boundary issues, shedding light on the need to develop regulation that protects both death doulas or those performing similar activities and those they engage with.

**Open Science registration:**

Https://osf.io/jkmsd.

## Plain language summary

Different perspectives on engaging with a death doula or someone who acts similarly to a death doula at the end of life

This systematic review explores the literature about different perspectives experiences when engaging with someone fulfilling the role of a death doula. Death doulas are non-medical but are concerned with the overall wellbeing of those who are dying. They have been gaining popularity as more people are unhappy with the medical care of those who are dying. In total, the researchers found 33 articles from 6 different countries that explored the experiences of dying people, family/caregivers, healthcare workers, and those in the death doula role themselves. This literature review found that death doulas can make sure that all parties have a generally more positive experience as someone nears the end of life. Simultaneously, death doulas are less likely to experience the negatives associated with traditional end-of-life care due to their flexibility, their unique way of creating relationships, and their ability to educate themselves and others on dying. However, there are some concerns about whether this role should be regulated for the safety of death doulas and those they care for. This research adds to the growing body of literature about death doulas by clarifying the experience of engaging with a death doula from multiple different perspectives, shedding light on the advantages of adding them to the care network when someone is dying.

## Introduction

The engagement of death doulas has become more common in response to increasing dissatisfaction with modern end-of-life care.^1^ In contrast to today’s system of medicine, for much of history, death was confined to the home and seen as natural. As people began living longer toward the end of the 19th century, medicine evolved, and dying happened more in hospitals,^2^ leading death care to become increasingly professionalized.^3^ In developed countries, this shift was met with increasingly expensive professional care, caregiver burnout, and increased emergency admissions in the last few days of life.^4^ With a medical system now more overwhelmed with complex end-of-life cases, providers are less able to provide individualized attention within acute care settings.^1^ The lack of resources contributes to dying people experiencing increased pain, anxiety, and dehumanization as they near death.^5,6^

A solution would seem to be to shift end-of-life care away from hospital settings. However, the medicalization movement has led to a sharp decrease in death literacy and an increase in the fear of death among informal caregivers, such as family members, who would be responsible for the patient if they were to move out of a formal hospital setting.^3^ While there is a demonstrated need for these informal caregivers, a need specifically highlighted during the Coronavirus pandemic,^7^ these caregivers not only feel unprepared but can experience extreme burdens or burnout when caring for someone with complex or terminal illness.^8^

As a result of displeasure with the medical system and an increase in informal caregiver burnout, people are beginning to reach for alternative methods of care.^4,9,10^ One such approach to death care is the concept of a death doula. The role of a death doula grew from the birthing doula movement, as both death and birth are natural human transitions that have been increasingly overmedicalized.^5^ Birth and death are times when people feel the need to plan to have a more transformative and personal experience; birth doula and death doula roles are both centered on facilitating the fulfillment of said plans.^9^

Defining the scope and role of death doulas can prove complicated, given their primary goal of meeting people’s needs and wishes at the end of life. Generally, those in a death doula role hold vigils, ease anxieties through meditative practices, promote death literacy to facilitate choice and uphold informed consent, organize legacy work, act as an advocate, mediate care coordination, and prepare both the dying person and their family for what is to come.^9–12^ They aim to provide non-medical physical, emotional, social, and spiritual support.^10,11,13–16^ Additionally, those acting as death doulas help to decrease caregiver burden by taking over caregiver roles.^8^

Currently, three reviews address the literature on death doulas. Two of these reviews focus on further clarifying and describing the role of the death doula.^17,18^ These reviews provide information on the range of death doula activities, how death doulas fit into the existing end-of-life and palliative care structure, role regulation, and the advantages and disadvantages of doula services. The most recent literature review on the topic expands on previous work by summarizing who is conducting research on death doulas and the research methodology used to do so, as well as the future directions posed by the available studies.^19^ In doing so, the authors draw two relevant conclusions about the genesis of this review. First, the authors conclude that the death doula role remains ambiguous and difficult to distinguish from other non-medical end-of-life roles that may not be designated under the title of death doulas (coordinators, volunteers, spiritual caregivers, etc.). Thus, the authors acknowledge the importance of future investigators taking a broad scope to gather more insights from those who may be doing this work. Second, the authors of this review, like the authors of the previous two reviews, call for an exploration of experiences from all stakeholders during death doula engagement, including dying people, family caregivers, healthcare workers, and the death doulas themselves. Therefore, this integrative review was conducted to synthesize the available literature on the experiences of engaging with death doulas or those who may be engaging in aligned activities from a broad range of perspectives. In this way, the emphasis is shifted from the naming convention, which is indeterminate and perhaps too exclusive, to an emphasis on activities being carried out regardless of what lay role the actor is in.

## Methods

### Literature review question

What are the experiences of dying individuals, family members/caregivers, healthcare workers, and death doulas (or those in activity-aligned roles) during engagement with such care and support?

### Design

This study had an explorative design, performed as an integrative review based on Whittemore and Knafl’s standards.^20^ This design was selected for this review as it allowed for the inclusion of both experimental and non-experimental data,^20^ making it amenable to a systematically conducted review concerning experience. Additionally, an integrative review was chosen as it extends beyond description, such as in a scoping review, instead exploring and interpreting the found evidence to produce findings as a synthesis. The steps of this review were based on Whittemore and Knafl’s suggestions for integrative reviews and included problem identification, literature search, data evaluation, data analysis, and presentation.^20^

### Literature search

In the problem identification stage, an adapted population, intervention, comparison, outcome (PICO) model was used to determine the population, phenomenon of interest, and context of the literature review, which were later integrated into the inclusion and exclusion criteria (Table 1). The inclusion criteria for the population of interest were broader than in previous reviews on death doulas. In the most recent literature review on death doulas, Thompson and Utz highlight the overlap between those who identify as death doulas and other non-medical supports at the end of life, such as care coordinators or navigators, volunteers, chaplains, etc.:

**Table 1.** Inclusion and exclusion criteria.

Concept	Inclusion criteria	Exclusion criteria
Phenomenon of interest	Experiences related to engagement of a death doula or those carrying out activities that align with the role of a death doula but are not otherwise named as a death doula (see population criteria)	Presentation of the role of death doula or related roles, without description of experience Focus on the care experiences of health or social care professionals
Population	Population includes either/or: • Death doulas and non-medical or lay personnel performing aligned activities	Birthing, labor, or maternal doulas Non-human death (e.g., animals or pets)
	including at least one of the following: ○ Holding vigils, promoting death literacy to facilitate choice and uphold informed consent, organizing legacy work, acting as an advocate, mediating care coordination, and preparing both the dying person and their family for what is to come, and otherwise providing non-clinical support • The dying person ○ Defined as having a life-limiting illness within the last 12 months of life • The family of the dying person • Health or social care professional providing care (where this relates to their experiences related to death doulas)	
Publication type	Empirical studies of any design, narrative pieces, or unpublished theses	Reviews, protocols, abstracts
Language	Full text available in English

Defining EOLD [end-of-life doula] broadly as providing specific kinds of non-medical support during EOL [end of life] (e.g., care coordinators) might allow future research to include broader insights from other non-medical support persons who do similar types of tasks, but who may not identify with the EOLD title. (Thompson and Utz,^19^ p. 12)

Thus, to include a breadth of insights not readily identified in previous literature reviews, the population was determined based on alignment with the general care activities provided by non-medical or lay personnel highlighted in previous reviews in studies such as: holding vigils, promoting death literacy to facilitate choice and uphold informed consent, organizing legacy work, acting as an advocate, mediating care coordination, and preparing both the dying person and their family for what is to come and otherwise providing non-clinical support. Since even those who identify as a death doula remain flexible in their chosen activities, those completing at least one of the pre-defined ‘death doula activities’ were included in this review to ensure complete capture of death doulas or those performing aligned activities. Additionally, inclusion criteria accounted for sources beyond empirical or scientific studies, such as narrative articles or stories, which increases the breadth of experiences captured in this review and responds to previous calls in other literature reviews for the inclusion of “gray literature” to increase experiential evidence.^19^

In the literature search stage, a comprehensive search of MedLine, CINAHL, and SCOPUS databases was conducted in September 2024. Databases were chosen at the suggestion of a specialist librarian for their inclusion of medical and anthropological sources, addressing the interdisciplinary nature of the death doula topic. An additional hand search of Lens.org was also recommended and utilized for an open-access, decolonized search of gray literature in addition to empirical studies. A search term strategy was developed in partnership with the specialist librarian from adapting the search terms of previous reviews on death doulas,^17,18^ as well as utilizing the Palliative cAre Literature rEview iTeraTive mEthod (PALETTE) framework.^21^ Additionally, the specialist librarian was consulted throughout the search strategy to ensure the search was capturing the highest yield possible while maintaining validity and accuracy.

Search terms were incorporated into a comprehensive search with the help of the specialist librarian. These searches differed slightly as EBSCO hosts (MedLine and CINAHL) employed controlled vocabulary such as MeSH headings, whereas SCOPUS and Lens.org did not (Appendix 1). Search terms that encompassed end-of-life and palliative care included hospice, terminal + illness or disease or cancer, and more (Appendix 1). Doula was searched for using terms like patient navigator, sitter, and companion, while a separate death doula-specific search was also performed, including other words for death doula, like thanadoula or amicus mortis (Appendix 1). The list of terms included in Appendix 1 is meant to be exhaustive and inclusive of roles that may carry out the activities of death doulas but that do not self-identify under the title of death doulas as referenced in previous literature reviews.^19^

Rayyan^22^ was used as a screening platform to ensure blind screens were completed by both authors. Fifty percent of the papers were double-screened during the initial title and abstract screening, while 20% of papers were double-screened during the full-text review. Both abstracts in the initial screening and full text in the subsequent screening were evaluated for inclusion based on the alignment with death doula activities, as determined in previous reviews, rather than solely based on the title of the role being described. After each review, results were unblinded in Rayyan so that discrepancies in screening could be seen. Discrepancies largely included whether experience was being described; one author may have opted to include an article that had a single reference to experience, while another may have excluded it for lack of experiential description. These disagreements were discussed until a consensus was reached between both authors. For included papers, reference lists and citations were scanned to identify potential papers.

### Quality appraisal

In the data evaluation stage, the Mixed Method Appraisal Tool (MMAT) was chosen as it allows for the evaluation of varying types of empirical data, inclusive of qualitative, quantitative, and mixed-method studies (Appendix 2).^23^ However, given that reports on experience, especially within a less-researched area such as this, tend to be anecdotal and fall under a gray literature category, this study was not limited to empirical research. Thus, it was decided early in the review process that, because experiences were being investigated, all literature from the final round of screening would be included regardless of quality score. Scores would instead correspond to the weight of each paper, which would correlate to the order they were analyzed and therefore a study’s contribution to theme creation and conclusions. The MMAT was therefore used despite not appraising gray literature, as all literature would be included with varying degrees of weight given to each journal’s overall contribution to findings and discussion. One author appraised all the papers, while a second author was given a random sampling.

### Data analysis

Due to the decision to include a wider range of literature as well as papers with mentions of experience in the context of death doula-aligned activities generally, it was recognized that all papers could not be given the same weight in the conclusions drawn. Thus, included papers were ordered, after full-text review, based on MMAT quality scores and evidence yield. High-yield papers referred directly to experiences as opposed to lower-yield papers, which detailed studies containing sporadic mentions of experience but did not have elucidating experience as their main purpose. In this manner, high-yield, high-quality papers were coded first and therefore given more weight as they qualified coding categories. Lower-quality, lower-yield paper was used as support for already identified codes and later for overarching themes rather than being determinative of theme in isolation.

Data extraction was first performed generally for author, year, country, aims, design, study population, and key findings relevant to experiences. Afterward, evidence was extracted using iterative coding, with unique codes being added as they were identified, and evidence being sorted into encompassing codes. Ultimately, each code was examined holistically for synthesis into descriptive and summative themes. Codes were verified by both authors, with one author coding all papers and another verifying using a random sampling. Themes were discussed collaboratively by both authors for total agreement.

## Findings

**Figure 1.**
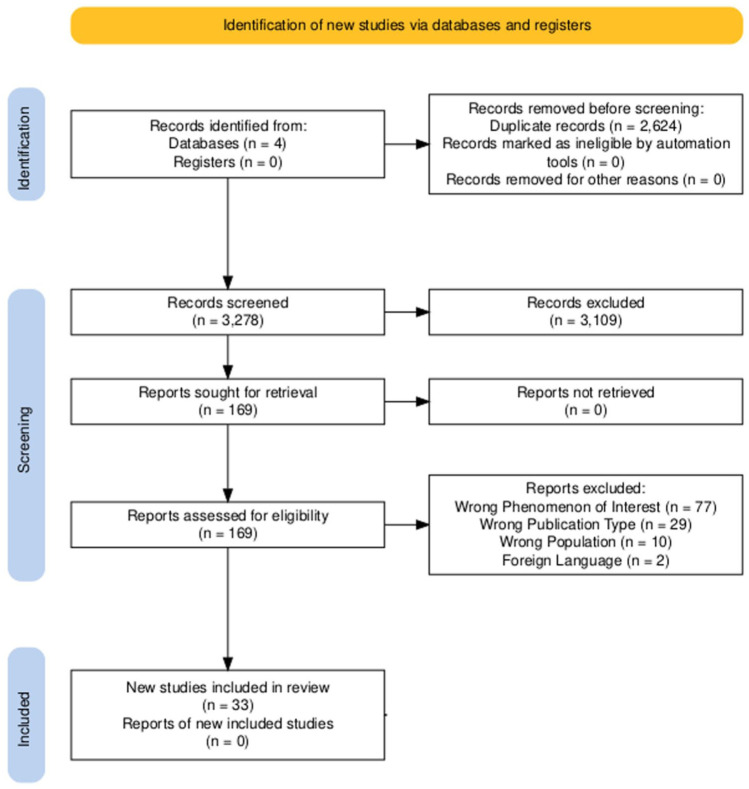
PRISMA diagram of studies.^23^

Thirty-three papers encompassing 25 individual studies were included and reported using PRISMA guidelines (Figure 1; Table 2).^24^ Papers came from six countries including the United States (*n* = 18), United Kingdom (*n* = 4), Australia (*n* = 5), Canada (*n* = 2), Singapore (*n* = 3), and China (*n* = 1). They encompassed a range of methods, including qualitative approaches (*n* = 20), mixed-methods (*n* = 7), quantitative approaches (*n* = 1), and non-empirical papers (*n* = 5). Most studies reported the perspective of those filling the role of a death doula or a role with aligned activities (*n* = 25), while others had study populations that included family members (*n* = 5), healthcare professionals (*n* = 5), and the dying persons themselves (*n* = 4). Although all evidence was included, papers with higher MMAT scores contributed more to theme designation and conclusions.

**Table 2.** Characteristics and summary of findings of included articles.

Author, year, country	Aims	Design	Study population	Key findings relevant to death doula engagement experience
Corporon, 2011, USA	- To describe the goals of Baylor’s death doula program - To elucidate the experiences of death doulas within Baylor’s program	Narrative article	Description of Baylor’s death doula program, including quoted participants in Baylor’s death doula program	- Death doulas describe interactions with patients as peaceful and happy, especially when facilitating spiritual requests
Germain et al., 2016, UK	To explore the experiences, perceptions, and motivations of volunteer end-of-life companions by phenomenologically analyzing reflective diary entries that were required of all volunteers in the pilot program	Thematic phenomenological analysis of reflective diary entries during a 26-week pilot implementation of a volunteer end-of-life “vigil” program	19 volunteers trained in non-medical care for the dying role	- Volunteers found themselves leaning on empathy and humility to move past their own emotions - Volunteers interpreted the process as iterative, knowing that they were constantly adjusting their approach based on past moments with those dying - Previous experience with loss affected the volunteers; positive experiences led to the desire to emulate good care/companionship, while negative experiences led them to want to improve end-of-life care for others - Reflective experiences allowed end-of-life volunteers to revisit their time with dying persons, either those with whom they volunteered or losses in their own life, and create new meaning from these situations - Volunteers devised individual ways to promote self-care and coping mechanisms while dealing with the challenging emotions that arose during their role
Goddard, 2016, USA	To explore the role of “transitional objects” in the dying process, specifically in dying processes in which an end-of-life doula or hospice care worker has been engaged	In-depth interviews and questionnaires with a “double hermeneutic” analysis design	8 end-of-life doulas and 4 hospice case workers	- Objects have helped end-of-life doulas carry out their role as objective companions in that it becomes a physical “in-between” - End-of-life doulas express feeling as though storytelling allows them to journey with the dying person and bring them to the point of transition - End-of-life doulas describe the experience of being a third party as being able to fulfill the need for someone to hold and respect a dying person and the family’s grief
Kaldy, 2016, USA	To describe the role and experiences of end-of-life doulas who are a part of INELDA	Newsletter reporting on end-of-life doulas	International End of Life Doula Association	- Families report a balance of fear/sadness with happiness/peace when working with an end-of-life doula - End-of-life doulas try to remain neutral even when there is fighting or emotion between parties present - Doctor reports feeling like their job is made easier when they have a doula to fall back on at the end of life, and that the doula can help doctors escape the feeling of inadequacy over not curing their patient
Trzeciak-Kerr, 2016, USA	- To understand the perception of end-of- life doulas and doulee family members’ lived experience while participating in an end-of-life doula program - Further elucidate how an end-of-life doula can add to existing end-of-life and palliative care structures	Qualitative interviews with existential phenomenological thematic analysis	15 “co-researchers” when referred to collectively; co-researchers comprised 11 end-of-life doulas and 4 doulee family members	- End-of-life doulas’ experience of “being there” for a patient means coming with no expectations or preconceived notions of what you “should” be doing - End-of-life doulas describe feeling intimacy and closeness despite remaining objective and malleable to dying persons’ needs/wishes - The presence of an end-of-life doula was reported to lessen the stress and guilt of family caregivers - End-of-life doulees’ family describes feeling as though their loved ones benefited from having someone to talk to who was not a familiar person - End-of-life doula process is iterative in terms of comfort level with death and dying, and while some report it “hurting,” many also report professional and personal growth through this burden of discomfort
Lentz, 2018, USA	To describe the role of a PCM team led by a palliative care doula	Narrative article	Narratives from 4 people who utilized the PCM team trained and led by a palliative care doula	- PCM team cites dying people finding peace through achieving their personal goals of care at the end of life - PCM team was able to act as a facilitator, resolving difficult patient/physician relationships - Family members report feeling like having someone “knowledgeable” reduced stress about end-of-life situations
Mitchell, 2019, Canada	To explore the role, services, and skills of death doulas operating in Saskatchewan	Qualitative interviews with an exploratory phenomenological approach	8 Saskatchewan death doulas	- Objectivity is required because death doulas have experienced families feeling disempowered if a death doula comes on “too strong” - Death doulas feel like they are “putting down roots” of education - Death doulas describe the experience of coming to this work through their helplessness and ignorance surrounding a death(s) that occurred in their lives - Death doulas express the need to have some “ease” when dealing with death in their own lives before they can help others - Self-care, in any capacity, was reported as important for maintaining objectivity and endurance
Bekelman et al., 2020, USA	To test the feasibility of a palliative care patient navigator program for Latino/a patients with advanced cancer	Ecological validity model and a participatory approach used to adapt an evidence-based counseling intervention + a pilot study to test the feasibility of said intervention	14 enrolled patients with advanced cancer in the pilot study arm who received non-medical care from patient navigators	- Patients’ experience with the navigators was overwhelmingly positive - Patients reported that having someone who listens and understands was most helpful
Fink et al., 2020, USA	- To understand the content of patient navigator meetings with intervention arm families - To evaluate the effect of lay patient navigators on patient experience	Qualitative analysis of documented field notes from a pilot study on lay patient navigator for Hispanic patients	Field notes from 4 Hispanic patient navigators—499 visits to 112 intervention families (families with a family member with advanced cancer)	- Patients felt like they were more able to handle conversations with healthcare professionals with the support of the navigator - Patients reported relief when working with the navigator on things like advanced care plans - Patients acknowledged that having the “strong” presence of someone else carried them through difficult times
Krawczyk and Rush, 2020, UK	To understand the development and practices of end-of-life doulas across four countries	Semi-structured interviews; abductive and iterative analysis approach with a narrative social constructionist framework	21 end-of-life doulas across the USA, Canada, UK, and Australia who completed interviews (1 incomplete)	- Many end-of-life doulas reported that having a healthcare or alternative medicine background or a personal experience with death (or both) drove them to this role - End-of-life doulas report that their experiences in their roles make them “change agents” for rethinking the current biomedical model of dying - Some end-of-life doulas report feeling most empowered in their role when they are no longer needed, and the families they work with can go out and replicate the work they did together - End-of-life doulas highlight the challenge of “therapizing” a family in grief, as end-of-life doulas are not necessarily trained or supported in this work
Rawlings et al., 2020, Australia	To clarify the ambiguity about the roles, experiences, skills, and education of death doulas	Online survey with multiple choice, Likert scales, or open-ended questions	85 people who self-identified as providing death doula engagement	- In death doulas’ opinions, economizing and registering death doulas would defeat the mission of accessibility inherent to the role - Death doulas believe that healthcare professionals are essential, but that death doulas can work in complement - Death doulas find that they are best acknowledged by healthcare professionals as an extra layer of care rather than replacing anyone’s role
Tumber, 2020, Canada	- To understand how death doulas fit into the larger framework of death care and death reform - To explore how beliefs concerning gender, death, and social privilege affect the death reform movement - To explore how death doulas may resist or uphold the commodification of their care work	Semi-structured interviews with a feminist phenomenological approach	6 death doulas	- In some death doulas’ experiences, starting to work with a family as soon as possible is important for building a relationship where death education can occur before death - Death doulas have stated that their experience working between all involved parties is closest to that of a mediator - There are past experiences where death doulas do not “stay in their lane,” which complicates their place in the fabric of palliative and end-of-life care as well as violates healthcare professionals’ trust - Death doulas show discomfort with being paid for their services, and those who describe this payment as a “sliding scale” or work for exchanges but feel in their experience that they wouldn’t be able to turn someone away who could not pay (moral economies)
Francis, 2021, USA	To explore the identities of end-of-life doulas through the lenses of gender, class, race, and occupational legitimacy	Semi-structured qualitative interviews with an iterative coding analysis	19 self-identifying death doulas who were pioneering the design of death doula courses	- Death doula often use intimacy to describe their experiences because it distinguishes it from being “personal” and therefore involving themselves - Death doula cites money transaction as a way to make engagement non-personal - There is a feeling among doula educators from their experience that personalization of care is intrinsic in that you can’t teach someone to just intuit what needs to be done - Some feel as though that a job well done means they no longer need to be a death doula, that is, everyone would be able to go back to this way of dying
Mallon, 2021, Australia	- To develop a model for a compassionate community network that expands the current model set forth by Abel et al. - To explore how perceived barriers and challenges of the death doula role could be addressed by a new compassionate community model, the Mallon model	Thematic analysis of research data from semi-structured interviews	28 death doulas from four different countries (Australia, Canada, the UK, and the USA)	- Some death doulas report that while relationships between themselves and the dying person or family are important, often it’s more about facilitating the creation or support of relationships within the dying person’s network and community - Death doulas’ experience of “slipping in and out” of roles is driven by the dying person’s changing wants and needs - Death doulas find that people’s fear/anxiety around helping a person who is dying can be alleviated through task-driven advice, that is, identifying something for them to do
Murphy, 2021, UK	- To describe the roles of end-of-life doulas, including training - To explore how advance care planning, legacy work, and do not attempt cardiopulmonary resuscitation issues are seen by end-of-life doulas - To understand how end-of-life doulas are integrated into the existing network of healthcare spaces at the end of life	Qualitative description	Narrative descriptions from the author and testimonials from 5 people who engaged with end-of-life doulas	- End-of-life doulas center the dying person and their family and then operate as a mediator for all go-betweens during the end-of-life experience - The dying person/family experience peace when an end-of-life doula can facilitate conversation where “nothing is off the table” - Families report information from end-of-life doulas, providing clarity in what would have been overwhelming and confusing - Families report that an end-of-life doula helped provide respite so that they could focus on loving their family member until the end
Rawlings et al., 2021, Australia	To gain the perspective of death doulas on their role within both health and social care environments	Semi-structured qualitative interviews with interpretive description analysis	A subgroup of 20 death doulas from a larger quantitative survey	- Death doula’s experience of advocating for the patient often feels more like translating between multiple parties - Sometimes, death doulas can feel unwelcome when working with healthcare professionals due to misconceptions about what they do - Other times, death doulas feel well-received by healthcare professionals who don’t have the time they would like to be with the dying person at the end of life - Some death doulas feel extremely uncomfortable with asking for money for their services, but it’s a gray area as they do give so much of themselves to those they are engaging in their services - Some death doulas are against regulation as they feel it would be restrictive to the flexible and personalized nature of their role
Dellinger Page et al., 2022, USA	- To record what the time an end-of-life doula spent with a dying person/family looked like - To explore the experiences of end-of-life doulas operating within family dynamics and attitudes toward death and dying	Mixed-methods approach consisting of a quantitative survey and qualitative interviews	Quantitative survey (*n* = 618 end-of-life doulas) and qualitative interviews with a subset from quantitative survey responders (*n* = 39 end-of-life doulas)	- End-of-life doulas feel as though the ability to provide peace and support is the greatest reward of engaging with dying people and their families - End-of-life doulas report that the “intensity” of their emotions is one of the biggest experiential challenges of their role - End-of-life doulas note financial aspects, such as whether they work for a price to be a prominent challenge - Even with the most emotionally complicated families, end-of-life doulas feel, in their experience, that they can help almost anyone find “one shared experience of love” with the dying person, which facilitates acceptance and more of a safer space to discuss death
Ellison, 2022, USA	- To share the experiences of an end-of-life doula who has worked with dying people who have intellectual disabilities - To explore how end-of-life doulas can better aid those with intellectual disabilities at the end of life	Narrative piece about the experience of an end-of-life doula	Author summary of available research relevant to death doulas engaging with people living with developmental or intellectual disability	- With the end-of-life doula as facilitator, family members’ “active” role in the dying process allows existing relationships with their loved ones to become more meaningful through and after death - End-of-life doulas who have worked with family members/disabilities have felt they must act more as a navigator between the health and disability sectors
Garces-Foley, 2022, USA	To understand the development of the non-medical end-of-life movement from end-of-life volunteers to professional end-of-life doulas	Exploratory in-depth interviews and analysis of print and online analysis	An undesignated number of non-medical end-of-life caregiver volunteers and supervisors at 12 sites; additional interviews with 8 people describing themselves as professional or aspiring professional end-of-life doulas or end-of-life midwives	- End-of-life volunteers speak about “life review” or the concept of experiencing and eliciting the dying person’s story so that they can feel as though it won’t be lost - End-of-life volunteers often experience the need to “fill the gap” or else fill in where care is lacking, either by families who are burdened by care or in healthcare settings—End-of-life vigilers experience honor in witnessing and aiding the transition from life to death
Hahn and Ogle, 2022, USA	To further elucidate the roles and motivations for end-of-life doulas	Semi-structured interviews; descriptive thematic analysis	12 certified end-of-life doulas from a larger study on end-of-life doulas	- End-of-life doulas speak to experiencing death in their personal life and feeling like their experiences as end-of-life doulas have given them a sense of grounding or control - Through experience, end-of-life doulas have found that personal emotional preparation is required to be able to then center the family/dying person’s emotions above their own - End-of-life doulas speak to the feeling of bringing peace and happiness to a dying person/family as a “gift” or “powerful” - There is a recognized need to be slightly removed from the emotional “quicksand” of the family or dying person, such that you can be fully present as an end-of-life doula
Chen and Yang, 2023, China	- To describe the experience of introducing hospice narrative doulas to a clinical hospice environment - To demonstrate how hospice narrative doulas can help people and their families at the end of life	Narrative article	Facilitator’s experience of introducing hospice narrative doulas to a hospice in China	- Before the utilization of hospice narrative doulas, patients felt more distressed by loneliness and isolation than fear of death itself - Hospice narrative doulas’ guidance through reflection on life stories can be seen to help dying people and families reinterpret death and its significance to them
DeDiego et al., 2023, USA	To understand the roles and experiences of death doulas operating in the USA	Concurrent triangulation mixed-methods design using surveys and video/audio file submissions	74 complete responses from USA-based death doulas	- Many death doulas felt called to the role after their own positive experiences with death doulas in their own time of need - Death doulas report a high level of compassion satisfaction – meaning made through supporting others—from their roles at the end of life - Death doulas report low levels of burnout
Dellinger Page and Husain, 2023, USA	To explore the different characteristics of end-of-life doulas certified by INELDA	Mixed-methods approach consisting of a quantitative survey and qualitative interviews	Quantitative survey (*n* = 618 end-of-life doulas) and qualitative interviews with a subset from quantitative survey responders (*n* = 39 end-of-life doulas)	- End-of-life doulas describe their experiences working within hospices as being a “bridge” between non-medical and medical parties - End-of-life doulas reported experiences of feeling as though they were distrusted by medical staff or felt there was a lack of communication with them as a team member - End-of-life doulas are fearful/wary of regulatory doula bodies - End-of-life doulas feel they are most sought out by those who had negative experiences with end-of-life care systems previously
Hahn et al., 2023, USA	To illuminate the challenges that face end-of-life doulas during end-of-life doula engagement	Semi-structured interviews; descriptive, thematic analysis	12 certified end-of-life doulas from a larger study on end-of-life doulas	- End-of-life doulas can find it difficult to create a boundary between being present and getting emotionally involved - End-of-life doulas often grieve both the loss of the dying person and the eventual loss of connection to their families - A lack of knowledge and death literacy is a main facilitator of fear and anxiety for families - End-of-life doulas can have to play mediator between the dying person, family members, healthcare professionals, etc.
Incorvaia, 2023, USA	- To describe the scope of practices, trainings, and functions of end-of-life doulas	Analytic autoethnography of two American end-of-life doula training programs	Two American end-of-life doula training programs using first-person narrative	- With so many doulas hailing from medical backgrounds, there is sometimes an expressed challenge in drawing a clear role boundary - The experience of being “seen and heard” by an objective end-of-life doula gives way to positive emotions - Being an end-of-life doula requires some intuitiveness and emotional maturity to be able to do what is individually right for every person or family engaged with; this can give end-of-life doulas in training anxiety - End-of-life doulas express the need to constantly reevaluate biases to remain objective with everyone they engage with
Krawczyk et al., 2023, UK	To summarize the findings, specifically relating to the experience of being a doula, from the first International End of Life Doula Symposium	Qualitative review of an international conference	40 people attended the International End of Life Doula Symposium in 2022	- Agreement in experience that death literacy increases calm during end-of-life experiences - Doulas’ experience of their role suggests that it is “heart work” and therefore shouldn’t be regulated, while others believe it is expert knowledge and should be paid for
Rawlings et al., 2023, Australia	- To understand the experiences of family members and patients who used a death doula for a dying loved one from the perspective of bereaved family members - To determine the benefits and challenges of death doula use To determine cultural shifts toward death and dying after death doula in terms of attitudes toward death and dying	Semi-structured interviews with an interpretative phenomenological approach	10 bereaved family members	- Death doula engagement allowed family members to feel prepared to deal with their loved one’s death from both an emotional, practical, and spiritual standpoint - Death doula helped the dying person feel empowered through the facilitation of personhood/autonomy - Death doula engagement empowered family members to want to help others who are struggling with the end of life
De Campos et al., 2024, USA	To determine the experience of hospice staff, patients, and end-of-life doula volunteers who participated in the implementation of a volunteer end-of-life doula pilot program within a hospice setting	Quantitative evaluations of experience during an end-of-life doula pilot program within a hospice	An unspecified number of hospice staff, caregivers as a proxy to patients, and end-of-life doula volunteers	- Caregivers felt that engaging with an end-of-life doula gave them more information about what to expect when their loved one was dying *and* made them feel more listened to than when working with just hospice staff
Donley and Fannin, 2024, USA	- To explore death doulas’ understanding of spiritual needs at the end of life and understand how they provide those needs - To understand the experience of death doulas who navigate working with those who have different spiritual or religious beliefs from them	Semi-structured qualitative interviews with deductive and inductive analysis	23 end-of-life doulas	- Many death doulas, after beginning their practice, decide to seek out additional training in diverse alternative care to personalize their engagement - Many death doulas feel compelled to be a guide through the “transition” they are witnessing - Some death doulas experience a struggle to be impartial when it comes to religious differences, while others believe death is equal for all and that relief is driven by being equipped with new knowledge
Rawlings et al., 2024, Australia	To understand healthcare practitioners’ perceptions of what a death doula is and does	Survey with some open text responses	317 healthcare professionals enrolled in the Dying2Learn Massive Open Online Course; *n* = 19 who practiced as a death doula or had an interest in practicing; *n* = 17 had professional experience as a death doula	- Healthcare professionals who identified as having worked alongside a death doula hold the work of the death doula in either positive regard due to “good effect,” or feel that they were entirely ineffective - There is some worry about the place for a death doula among healthcare professionals and the network of palliative care - Some healthcare professionals feel offended by the offer of a death doula, as they see it as a failure on their part to do their work
Tay Ying, 2024, Singapore	To describe the experiences of one death doula working in Singapore	Autobiographical narrative story	Written by a death doula	- Death doula describes coming to end-of-life doula work as a “calling” - Death doula work can be slow, as it often becomes just as much about life planning as end-of-life planning
Yoong et al., 2024, Singapore	To elucidate nursing students’ experience of a death “service-learning program” during a 6-month palliative care service rotation	Semi-structured focus group discussion with descriptive qualitative analysis	14 nursing students who had participated in a 2-day death doula course	- Death doula student trainees felt unexpected gratitude at being witnesses during the end of life and felt that death doula training eased some of their fears and misconceptions surrounding death and dying - Death doula student trainees recognized that operating as death doulas allowed them to practice person-centered care that they do not always feel they can do as nurses - Recognized need for death doula-like positions to help ease societal taboos around death and dying
Yoong et al., 2024, Singapore	- To evaluate the impact of a death doula service-learning experience for nursing students on students’ competencies in palliative care - To understand the experiences of nursing students enrolled in the service-learning death doula program	Mixed-methods approach using a randomized controlled trial and thematic analysis of pre- and post-service-learning reflections	16 nursing students enrolled in the intervention arm; 17 students enrolled in the control group	- Student death doulas showed improved attitudes toward death after completion of training - Service-learning helped students have a more positive regard toward palliative patients - Student death doulas report that service learning was rewarding as they had a positive impact on patients at the end of life - Student death doulas report that their experiences demonstrate the need for a death doula to have emotional control when working in this role

PCM, palliative care ministry; UK, United Kingdom; USA, United States.

Six themes resulted from careful analysis and synthesis: emotions before and after engagement, transforming fear through knowledge and literacy, objective companionship, the death doula as a mediator, the death doula cycle, and the tension between flexibility and regulation.

### Emotions before and after engagement

A range of emotions was experienced by death doulas or those performing aligned activities. Positive emotions included peace and joy at being able to fulfill the wishes of families and dying people,^25–29^ as well as feelings of pride and honor at being present during such a vulnerable time.^28,30–32^ They also experienced negative emotions like stress, sadness, role anxiety, or more existential emotions about death. These emotions required them to utilize self-care practices to decrease burnout.^26,28,33,34^ Negative emotions also came from processing grief over the loss of the dying person and the future loss of the connection they had cultivated with the family.^35–37^ The acute intensity of these negative emotions is emphasized as one of the largest challenges of being a death doula or performing aligned activities and highlights the importance of coping mechanisms and emotional control.^27,31^ However, positive emotions associated with performing death doula-aligned activities facilitate extreme role fulfillment, which decreases emotional burnout.^27,31,37^

For families and dying people (doulees), engaging a death doula or someone performing aligned activities led to a transformation of negative to positive emotions. Before engagement, doulees felt fear and anxiety, due to uncertainty about the healthcare system and death.^38,39^ Being on the receiving end of the death doula-aligned activities transformed negative emotions into peace and happiness, or general positivity.^38–40^ Engagement in these activities alleviated the burden often associated with being a family caregiver.^41,42^ One family doulee described her and her dying husband’s experience:

First of all, she gave me time and it helped relieve me of the anxiety of being with him. . . It gave him a peace of mind. . .I think that was very important, and, to me as well. I think it was great for him because whatever they discussed, whatever transpired, was helpful for him and for me as well. (Trzeciak-Kerr,^42^ p. 104)

As a whole, death doulas and those performing similar activities perceived that the respite that they provided helped to facilitate more feelings of closeness and love between loved ones and dying people.^43^ Dying people themselves reported that their distress primarily comes from fear of loneliness,^44^ but, based on reports from death doulas or those in activity-aligned roles, they later felt more seen and heard, generating positive emotions.^34^ These positive emotions contributed to feeling more empowered and autonomous in their dying process.^36^

### Transforming fear through knowledge and death literacy

Doulees emphasized that it was the death doula or those in activity-aligned roles’ knowledge of the medical system, the dying process, and what happens after that alleviated negative emotions.^35,36,39,43,45–47^ Family members felt more purpose when death doulas or those performing similar activities translated their knowledge about death into actionable items that made a hard-to-navigate system easier.^48,49^ In the words of a family caregiver:

It was a very confusing time for myself and my mother. The comprehensiveness of the information and the sympathetic way that (the end-of-life doula) provided it was hugely refreshing for all of us involved and gave us a lot more clarity. (Murphy,^43^ p. 337)

Anxiety also stemmed from the “unknowns” of death and the taboo of discussing dying more openly; feelings of peace were facilitated through open conversation based on the knowledge and experiences of death doulas or those in activity-aligned roles.^27,36,43^

Similarly, death doulas or those performing similar activities felt that relying on facts relieved concerns about effectiveness in their roles.^33^ Training that emphasized knowledge and death literacy enabled those performing death doula-aligned activities to feel more comfortable discussing death.^31^ Often, their previous experiences in healthcare or with personal losses fueled a search for more knowledge, allowing them to eventually step into the role of death doula or a role carrying out similar activities.^33,34,50^

### Objective companionship

One of the commonly reported challenges that death doulas or those in activity-aligned roles experienced was how to establish a relationship built on intimacy and impartiality simultaneously.^26–29,33–36,40,42,47,51,52^ We came to refer to this theme as objective companionship – a relationship that is intimate but not “personal,” because death doulas or those performing similar activities felt most effective when their own emotions were removed and they could create bonds through empathy and listening.^26,52^ A death doula says:

I’m sitting next to you, and you’re in your quicksand. But I’m not going to get in your quicksand. And it’s that thin veil that you put between you and your patient . . . I am not turning away like I am in this with you. But my emotions aren’t in there. (Hahn and Ogle,^28^ p. 1620)

Establishing an objective companionship successfully allowed doulees to experience a sense of openness and comfort unencumbered by the worry of being a burden.^27,40,42^ If death doulas or those in activity-aligned roles over-expressed their opinions, families reported feeling overwhelmed and disenfranchised from their experiences of the dying and bereavement process.^33,34,51^ Overall, there was agreement in the literature concerning the need for objectivity, with some variance on whether maintaining relationships with family members after a doulee’s death was appropriate.^35,36^

Some death doulas and those performing similar activities found that adding a transactional layer, like payment, helps to ensure objective companionship.^52^ Others experienced a visceral distaste for payment as they believed that it would make their services inaccessible and detract from the intimacy of the role.^27,45,47,53,54^ Generally, death doulas or those in activity-aligned roles seemed to be searching for an in-between where a “moral economy” can be established, such that they resolve the turmoil over the need to make a living and the altruism that is at the heart of the role.^47,54^

### Death doula as a mediator

Death doulas or those performing similar activities used the term mediator to describe their experiences of working within different areas of the end-of-life care network.^35,43,47,54,55^ In the relationship between healthcare professionals and families, death doulas and those in activity-aligned roles provided an extra layer of care, which sometimes alleviated professionals’ feelings of failure when they lacked time to be with the family.^30,31,38,53,54,56^ Families also reported that having a death doula or someone performing similar activities to assist in navigating the medical system and resolving tense relationships with healthcare professionals provided relief.^39,41,49^

In their position as mediators within the healthcare system, death doulas or those performing similar activities felt some healthcare professionals did not take their work seriously and undermined their goals of care.^50,54–56^ This may stem from healthcare professionals feeling inadequate when observing a death doula or those in activity-aligned roles’ successes.^56^ Conversely, if death doulas or those performing aligned activities stepped out of the role as a mediator into a clinical area, healthcare professionals lost trust, and the scope of activities of those in lay roles at the end of life became unclear.^34,47,54^

As a mediator between family members and the dying person, a death doula and those performing similar activities described a neutral stance if there is infighting.^38^ They experienced success when facilitating the sharing of “experiences of love” rather than tackling complex dynamics.^27,44^ They have also experienced that acting as a third party to create compassionate community networks can help address doulees’ needs on a larger level by connecting families and dying people with assistance in their communities.^48^

Death doula and those in activity-aligned roles also had the experience of being a mediator between life and death.^29,51^ One death doula described this phenomenon:

Death is like a colleague with another company. . .But we sort of work together. I don’t know what she does, and she doesn’t necessarily get involved with what I do. But my job is to help people have a good transition. (Donley and Fannin,^29^ p. 12)

Activities such as facilitating legacy work, providing spiritual guidance, hearing stories before death, and retelling stories after death allowed death doulas and those performing similar activities to occupy a space between life and death.^30,42,44,51^

### The death doula cycle

Death doulas or those in activity-aligned roles described engagement as cyclical in that families wanted to share what they gained from their experience with others who were struggling, effectively passing on the role of a death doula or someone performing similar activities.^36^ This is evidenced by the death doula or those in aligned activities across the included studies who cited their own experiences being a part of someone’s dying process as a reason for seeking out the role.^26,28,33,37,50,53,57^ Performing these activities “provided [doulas or those in activity-aligned roles]. . .a revised and broader context to explore their personal experiences of bereavement and loss, allowing them to gain new understanding and develop new meaning” (p. 6).^26^ This continued reflection also revealed the need for continued education as death doulas and those performing similar activities identified holes in their capabilities based on their experiences of previous engagements.^29,32^

### Tension between flexibility and regulation

Death doulas or those in activity-aligned roles found that being completely open and malleable to the wants and needs of everyone they engage with not only separates them from the other roles in the death care network but also accounts for their success.^34,42,48,50,52,55^ One death doula described this flexibility:

So, in terms of . . . slipping in and out of various roles . . . it’s really patient-driven, and I’d much rather it be that way in the first place . . . it’s really about the care that they [clients] need at that moment. (Mallon,^48^ p. 8)

Since the work they do is with a vulnerable population, some death doulas and those performing similar activities believe that the role should be regulated and governed.^45^ This regulation, however, is believed to decrease the degree to which those performing death doula-aligned activities can be flexible and thereby hinder both the experience of those performing these activities and the experience of the families involved.^54,55^

## Discussion

### Summary of main findings

In summary, the aim of this review was to explore the experiences of all stakeholders during the engagement of death doulas and those in activity-aligned roles. Ultimately, the analysis of included papers yielded six themes that encapsulate the experiential evidence of the included studies: emotions before and after engagement, transforming fear through knowledge and literacy, objective companionship, the death doula as a mediator, the death doula cycle, and the tension between flexibility and regulation. It should be noted that the following discussion and conclusions are suggested on the basis of limited perspectives from dying persons, families, and health and social care workers, with the majority of the included papers being from the perspective of death doulas or those in an activity-aligned role.

Evidence from the review suggests that during engagement with a death doula or someone performing similar roles, dying people and their families experienced a shift from negative to positive emotions, formed a unique bond with those performing death doula-aligned activities based on trust, and gained knowledge relating to the dying process that often encouraged others to perform similar activities in the future. In witnessing this relationship between their patients and a doula or someone in an activity-aligned role, some healthcare workers felt relieved and more able to perform their medical duties, knowing that a doula is taking care of the patient’s psychosocial well-being. Other times, healthcare workers felt their position was being encroached upon, likely due to the ill-defined regulation of death doulas and those performing similar roles. Those performing these activities themselves experienced a range of emotions from anxiety at performing their role and caring for themselves to extreme fulfillment and honor at being present for a dying person and their family. Death doula and those in activity-aligned roles also echoed the unique experience of creating a relationship with those they engage; however, they emphasized the importance of finding objectivity in their work. While death doula or those in activity-aligned roles in several studies reported the experience of being called to this role through their own experiences with death or health, others struggled with establishing it as a “job” rather than just a calling, leading to concerns about payment and regulation.

### Comparison to wider literature

The shift between negative to positive emotions described by both dying people and families seems to center around an alleviation of guilt and burden. Research on caregiver burden has described negative emotions related to an inability to meet the needs of a seriously ill person and difficulty navigating the medical care system, which mirrors the negative emotions reported by families who are informally caring for the dying before engaging a death doula or someone performing similar activities.^58,59^ However, the addition of a death doula or someone in an activity-aligned role to the care network of a dying person not only relieves these emotions but also allows for positive emotions, such as closeness and love, to replace them. Death doulas and those in activity-aligned roles may provide an answer to the resounding call for inclusion of caregiver burden and burnout in the holistic treatment plan of a dying person,^58,60^ not only because they facilitate this transformation of emotions but because they do not seem to be affected by the same burnout despite the burden of care being passed to them.^37^ A possible reason for this separate experience may be how death doulas or those performing similar activities experience extreme role fulfillment. Literature on care burnout in healthcare workers indicates that a lack of fulfillment, usually resulting from poor organizational structure and support, increases burnout significantly.^61^ Oppositely, the fulfillment described by death doulas and those in activity-aligned roles, which seems to relate to the flexibility of these roles, may work to combat burnout typically experienced by formal and informal caregivers.

Another explanation for the absence of burnout experienced by death doulas and those performing similar activities seems to rely on the concept of objective companionship as established by this review. Establishing a space of closeness while remaining impartial is a unique hallmark of those who practice these death doula-aligned activities. Literature reviews on the nurse–patient relationship indicate that conditions such as time constraints, environmental factors, communication barriers, and stress of the system contribute to a lack of trust within the healthcare worker and patient relationship.^62^ The flexibility provided by the role allows for death doulas and those performing similar activities to be present for any need of the patient, thus facilitating trust more readily than a traditional healthcare role. However, the cultivation of such a relationship may risk a violation of boundaries. Ethical research into therapeutic relationships similar to that between families or a dying person and a death doula or those performing similar activities reveals that a violation of boundaries, such as the therapist oversharing or imbuing too many personal emotions, can invalidate the client’s experience.^63^ To avoid boundary-crossing, therapeutic research reveals that communication of boundaries is necessary to remain open and empathetic within such a relationship.^63^ While this research affirms the need for objective companionship, it also emphasizes the existing argument for regulation for death doulas or those in activity-aligned roles so that boundaries can be less subjective and both doula and doulee are more protected.

### Strengths and limitations

This review is the first to explore the experience of engaging with a death doula or those performing similar roles. As such, a choice was made to keep the population of this review specifically broad while the phenomenon of interest, experience, was narrow. In doing so, experience could be investigated from a breadth of perspectives, painting a more complete picture of engagement with someone performing death doula-aligned activities. By elucidating experience from all perspectives, inclusive of death doulas or those in activity-aligned roles themselves, more was revealed about the roles of those performing these types of activities and their place within the end-of-life care network. Another strength of this review is the use of broader inclusion criteria concerning search terms for “death doula” to account for previously excluded voices of those whose activities are similar to death doulas but who do not use the title of death doula.^19^ As previously acknowledged by existing literature reviews on this topic, the role of a death doula is still being clarified, and conversations are presently ongoing about whether to develop a unifying certification for death doulas such that there is a single definition. Instead of potentially excluding voices due to the lack of consensus on a single “death doula” definition, this review includes all those who work on activities that are aligned with the activities carried out by death doulas, as determined by the previous literature review.

Given that death doulas and those performing similar activities are a recent development within end-of-life care, the research specifically exploring experience was limited, and much of it was informal and anecdotal. Thus, the decision was made to include gray literature from a limited hand search to better encompass available information on the experience of engaging with a death doula or those in activity-aligned roles. While our interpretation of data gave more weight to empirical studies, our findings should be considered in the context of including low-quality literature. However, given our choice to order literature based on experiential evidence yield and MMAT quality scores, only empirical and relevant studies had the highest weight in theme creation. While it is possible that there was some bias involved in deciding the order the studies would be analyzed, author consensus on the order of analysis was achieved to address this possible limitation. Additionally, given our effort to diminish the risk of alienating key perspectives, it is possible that we were too broad in our inclusion of who could be considered to be acting in activity-aligned roles. This limitation, however, highlights again the pressing need for an agreed-upon definition such that research isn’t confounded or diminished by the inclusion or exclusion of certain non-medical end-of-life care roles. Lastly, while our search terms encompassed “death doula” to the best of our knowledge, it is acknowledged that death doula-like roles are likely performed in non-Western societies under different names, which limited the geographical reach of this study to mostly Western regions. It is possible that the use of comprehensive databases—MedLine, CINAHL, and SCOPUS—missed literature that could be found in other smaller and specific databases. However, an open-access, decolonized hand search of Lens.org, at the recommendation of a specialist librarian, was conducted to mitigate the effects of limitation.

### Recommendations for policy, practice, and research

Recommendations for policy, practice, and research are based on the evidence presented in this review. Based on this body of literature, policy surrounding death doulas and those in activity-aligned roles must be approached cautiously. While ethical boundary setting within the doula–doulee relationship seems necessary, there is a call against regulation within the community itself for fear of limiting the flexibility that makes the role unique and successful. It may be helpful to consider registration or certification under a united body for organizational and data purposes before implementing a policy concerned with regulation. Furthermore, as discovered during the construction of this review, there is a need for continued clarification of who can be considered a death doula and whether the activities attributed to death doulas by previous reviews can be applied to other non-medical supports at the end of life, regardless of self-identification. This action is increasingly important in terms of conducting rigorous research in this field that does not exclude necessary voices or include too wide a range.

In terms of practice, this review highlights the numerous ways that death doulas and those performing similar activities add to the team of professionals who work within end-of-life care. The flexibility, both in terms of time and role, allows death doulas and those in activity-aligned roles to close gaps in end-of-life care that have been cited by patients, families, and healthcare workers. However, there is still resistance to the role in some healthcare spaces, mostly because of misunderstandings of role boundaries. This is another way certification under a registered organization could aid this work; by legitimizing the role, healthcare workers may feel their jobs are less threatened and be more inclined to work with death doulas or those performing similar activities.

The objective of this paper was to explore the experiences of engaging with death doulas or those performing aligned activities from multiple perspectives, including the dying person, their families, health and social care professionals, and death doulas or those in activity-aligned roles themselves. While this objective was met, when examining the body of included literature in this review, only 4 out of 33 included the perspective of the dying person, 5 included the perspective of the family, and 5 included the perspective of health or social care professionals. Thus, these voices begin to add to the larger discussion on death doulas and other activity-aligned roles, but future research must be directed toward the experience of people who are actively utilizing death doulas or those performing similar activities. The precarious nature of this population, in that they are all at the end of life, has led to a dearth in this perspective. It is important to understand how this type of engagement facilitates or creates boundaries to the end-of-life process for those who are dying instead of through family proxies.

## Conclusion

Engaging with a death doula or someone in an activity-aligned role at the end of life has been demonstrated to be one way that dying people, family caregivers, and healthcare workers can address the issues facing end-of-life care currently. Due to limited literature from perspectives outside of the death doula or those performing similar activities, it is suggested that engaging with those in a death doula or activity-aligned role provides a unique and positive experience for all those involved, providing strengthened evidence to continue exploring the inclusion of those engaging in death doula or activity-aligned roles within the end-of-life care network.We propose, based on the evidence in this review, that death doulas and those performing similar activities’ have a degree of flexibility that allows them to facilitate a more positive experience for the patient, family caregiver, and healthcare workers. This flexibility seemingly allows them to work on many levels, including increasing death literacy, reducing negative emotions, and acting as a mediator. However, it is this same flexibility that seems to cause the role confusion that makes engagement with a death doula or those in an activity-aligned role mystifying for healthcare workers, and previous research on the subject. Thus, a future challenge to be tackled by stakeholders in this field is to regulate the role in a manner that doesn’t decrease the positive experience of engagement but that clarifies the role to ensure clarification and protection for all involved. Additionally, future research must continue to investigate the experience of engaging with a death doula or those performing similar activities, specifically from the perspective of the dying person, to continue to show the need to include death doula and those in activity-aligned roles more readily within end-of-life care systems and to educate a wider audience about a resource they could reach in an end-of-life care setting.

## Appendix

**Appendix 1.** Search terms and strategy.

Topic	Keywords			
End of life/death/palliative care (S1)	EBSCOHOST: MH ( (“Palliative Care”) OR (“Palliative Medicine”) OR (“Hospice and Palliative Care Nursing”) OR (“Terminal Care”) OR (“Hospice Care”) ) OR TI ( ( palliative OR dying OR death OR bereave* OR (end* N3 life) OR end-of-life OR “advanced care” OR hospice* OR (terminal* N3 (diseas* OR ill* OR cancer*)) OR (advanced N3 (diseas* OR ill* or cancer*)) ) OR AB ( ( palliative OR dying OR death OR bereave* OR (end* N3 life) OR end-of-life OR “advanced care” OR hospice* OR (terminal* N3 (diseas* OR ill* OR cancer*)) OR (advanced N3 (diseas* OR ill* or cancer*)) ) SCOPUS: Article Title, Abstract, Key Words: “Palliative Care” OR “Palliative Medicine” OR “Hospice and Palliative Care Nursing” OR “Terminal Care” OR “Hospice Care” OR palliative OR dying OR death OR bereave* OR (end* W/3 life) OR end-of-life OR “advanced care” OR hospice* OR (terminal* W/3 (diseas* OR ill* OR cancer*)) OR (advanced W/3 (diseas* OR ill* or cancer*))			
Doula (S2)	EBSCO: MH doulas OR TI ( doula* OR “lay navigator” OR “patient navigator” OR ((death OR dying OR soul OR mourn* OR transition OR end-of-life OR bardo) N3 (facilitator OR midwife OR advocate OR advisor OR consultant OR sitter OR walker OR guide* OR coach* OR companion* OR keeper* OR attendant* OR mentor*)) ) OR AB ( doula* OR “lay navigator” OR “patient navigator” OR ((death OR dying OR soul OR mourn* OR transition OR end-of-life OR bardo) N3 (facilitator OR midwife OR advocate OR advisor OR consultant OR sitter OR walker OR guide* OR coach* OR companion* OR keeper* OR attendant* OR mentor*)) ) SCOPUS: Article Title, Abstract, Key Words: doulas OR doula* OR “lay navigator” OR “patient navigator” OR (death OR dying OR soul OR mourn* OR transition OR end-of-life OR bardo) W/3 (facilitator OR midwife OR advocate OR advisor OR consultant OR sitter OR walker OR guide* OR coach* OR companion* OR keeper* OR attendant* OR mentor*)			
Death doula—Specific (S3)	AB ( stervensbegeleiding OR thanadoulas OR thanadoula OR Psychopomp* OR “amicus mortis*” ) OR TI ( stervensbegeleiding OR thanadoulas OR thanadoula OR Psychopomp* OR “amicus mortis*” ) SCOPUS: ( stervensbegeleiding OR thanadoulas OR thanadoula OR Psychopomp* OR “amicus mortis*” )			
Lens.org hand search	Title: ( death AND doula ) OR ( Abstract: ( death AND doula ) OR ( Full Text: ( death AND doula ) OR ( Title: ( end AND ( of AND ( life AND doula ) ) ) OR ( Abstract: ( end AND ( of AND ( life AND doula ) ) ) OR ( Full Text: ( end AND ( of AND ( life AND doula ) ) ) OR ( Title: ( end-of-life AND doula ) OR ( Abstract: ( end-of-life AND doula ) OR ( Full Text: ( end-of-life AND doula ) OR ( Title: ( amicus AND mortis ) OR ( Abstract: ( amicus AND mortis ) OR ( Full Text: ( amicus AND mortis ) OR ( Title: thanadoula OR ( Abstract: thanadoula OR Full Text: thanadoula ) ) ) ) ) ) ) ) ) ) ) ) )			
Searches	Databases and results			
	MedLine	CINAHL	SCOPUS	Lens.org
S1	1,371,452	337,968	2,212,400	
S2	4081	3353	5787	
S3	7	1	76	
S1 AND S2 = S4	1875	1134	2456	
S3 OR S4	1882	1135	2531	
				354

**Appendix 2.**

Author(s), year, country	Study design	Aims	Key findings relevant to death doula engagement experience	Mixed Methods Appraisal Tool (MMAT) V2018
Corporon, 2011, USA	Narrative article	- To describe the goals of Baylor’s death doula program - To elucidate the experiences of death doulas within Baylor’s program	- Death doulas describe interactions with patients as peaceful and happy, especially when facilitating spiritual requests	–
Germain et al., 2016, UK	Qualitative—phenomenology	To explore the experiences, perceptions, and motivations of volunteer end-of-life companions by phenomenologically analyzing reflective diary entries that were required of all volunteers in the pilot program	- Volunteers found themselves leaning on empathy and humility to move past their own emotions - Volunteers interpreted the process as iterative, knowing that they were constantly adjusting their approach based on past moments with those dying - Previous experience with loss affected the volunteers; positive experiences led to the desire to emulate good care/companionship, while negative experiences led them to want to improve end-of-life care for others - Reflective experiences allowed end-of-life volunteers to revisit their time with dying persons, either those with whom they volunteered or losses in their own life, and create new meaning from these situations - Volunteers devised individual ways to promote self-care and coping mechanisms while dealing with the challenging emotions that arose during their role	1. Is the qualitative approach appropriate to answer the research question? **Y** 2. Are the qualitative data collection methods adequate to address the research question? **Y** 3. Are the findings adequately derived from the data? **Y** 4. Is the interpretation of results sufficiently substantiated by data? **Y** 5. Is there coherence between qualitative data sources, collection, analysis, and interpretation? **Y**
Goddard, 2016, USA	Qualitative—phenomenology	To explore the role of “transitional objects” in the dying process, specifically in dying processes in which an end-of-life doula or hospice care worker has been engaged	- Objects have helped end-of-life doulas carry out their role as objective companions in that it becomes a physical “in-between” - End-of-life doulas express feeling as though storytelling allows them to journey with the dying person and bring them to the point of transition - End-of-life doulas describe the experience of being a third party as being able to fulfill the need for someone to hold and respect a dying person and family’s grief	1. Is the qualitative approach appropriate to answer the research question? **Y** 2. Are the qualitative data collection methods adequate to address the research question? **Y** 3. Are the findings adequately derived from the data? **Y** 4. Is the interpretation of results sufficiently substantiated by data? **Y** 5. Is there coherence between qualitative data sources, collection, analysis, and interpretation? **Y**
Kaldy, 2016, USA	Newsletter reporting on end-of-life doulas	To describe the role and experiences of end-of-life doulas who are a part of INELDA	- Families report a balance of fear/sadness with happiness/peace when working with an end-of-life doula - End-of-life doulas try to remain neutral even when there is fighting or emotion between parties present - Doctor reports feeling like their job is made easier when they have a doula to fall back on at the end-of-life, and that the doula can help doctors escape the feeling of inadequacy over not curing their patient	–
Trzeciak-Kerr, 2016, USA	Qualitative—phenomenology	- To understand the perception of end-of-life doulas and doulee family members’ lived experience while participating in an end-of-life doula program - Further elucidate how an end-of-life doula can add to existing end-of-life and palliative care structures	- End-of-life doulas’ experience of “being there” for a patient means coming with no expectations or preconceived notions of what you “should” be doing - End-of-life doulas describe feeling intimacy and closeness despite remaining objective and malleable to dying persons’ needs/wishes - The presence of an end-of-life doula was reported to lessen the stress and guilt of family caregivers - End-of-life doulees’ family describe feeling as though their loved ones benefited from having someone to talk to who was not a familiar person - End-of-life doula process is iterative in terms of comfort level with death and dying, and while some report it “hurting,” many also report professional and personal growth through this burden of discomfort	1. Is the qualitative approach appropriate to answer the research question? **Y** 2. Are the qualitative data collection methods adequate to address the research question? **Y** 3. Are the findings adequately derived from the data? **Y** 4. Is the interpretation of results sufficiently substantiated by data? **Y** 5. Is there coherence between qualitative data sources, collection, analysis, and interpretation? **Y**
Lentz, 2018, USA	Qualitative article	To describe the role of a PCM team led by a palliative care doula	- PCM team cites dying people finding peace through achieving their personal goals of care at the end of life - PCM team was able to act as a facilitator, resolving difficult patient/physician relationships - Family members report feeling like having someone “knowledgeable” reduced stress about end-of-life situations	–
Mitchell, 2019, Canada	Qualitative—phenomenology	To explore the role, services, and skills of death doulas operating in Saskatchewan	- Objectivity is required because death doulas have experienced families feeling disempowered if a death doula comes on “too strong” - Death doulas feel like they are “putting down roots” of education - Death doulas describe the experience of coming to this work through their helplessness and ignorance surrounding a death(s) that occurred in their lives - Death doulas express the need to have some “ease” when dealing with death in their own lives before they can help others - Self-care, in any capacity, was reported as important for maintaining objectivity and endurance	1. Is the qualitative approach appropriate to answer the research question? **Y** 2. Are the qualitative data collection methods adequate to address the research question? **Y** 3. Are the findings adequately derived from the data? **Y** 4. Is the interpretation of results sufficiently substantiated by data? **Y** 5. Is there coherence between qualitative data sources, collection, analysis, and interpretation? **Y**
Bekelman et al., 2020, USA	Mixed-methods approach—sequential explanatory design	To test the feasibility of a palliative care patient navigator program for Latino/a patients with advanced cancer	- Patients’ experience with the navigators was overwhelmingly positive - Patients reported that having someone who listens and understands was most helpful	1. Is there an adequate rationale for using a mixed-methods design to address the research question? **Y** 2. Are the different components of the study effectively integrated to answer the research question? **Y** 3. Are the outputs of the integration of qualitative and quantitative components adequately interpreted? **Y** 4. Are divergences and inconsistencies between quantitative and qualitative results adequately addressed? **Y** 5. Do the different components of the study adhere to the quality criteria of each tradition of the methods involved? **Y**
Fink et al., 2020, USA	Qualitative description	- To understand the content of patient navigator meetings with intervention arm families - To evaluate the effect of lay patient navigators on patient experience	- Patients felt like they were more able to handle conversations with healthcare professionals with the support of the navigator - Patients reported relief when working with the navigator on things like advanced care plans - Patients acknowledged that having the “strong” presence of someone else carried them through difficult times	1. Is the qualitative approach appropriate to answer the research question? **Y** 2. Are the qualitative data collection methods adequate to address the research question? **Y** 3. Are the findings adequately derived from the data? **Y** 4. Is the interpretation of results sufficiently substantiated by data? **Y** 5. Is there coherence between qualitative data sources, collection, analysis, and interpretation? **Y**
Krawczyk and Rush, 2020, UK	Qualitative description	To understand the development and practices of end-of-life doulas across four countries	- Many end-of-life doulas reported that having a healthcare or alternative medicine background or a personal experience with death (or both) drove them to this role - End-of-life doulas report that their experiences in their roles make them “change agents” for rethinking the current biomedical model of dying - Some end-of-life doulas report feeling most empowered in their role when they are no longer needed, and the families they work with can go out and replicate the work they did together - End-of-life doulas highlight the challenge of “therapizing” a family in grief, as end-of-life doulas are not necessarily trained or supported in this work	1. Is the qualitative approach appropriate to answer the research question? **Y** 2. Are the qualitative data collection methods adequate to address the research question? **Y** 3. Are the findings adequately derived from the data? **Y** 4. Is the interpretation of results sufficiently substantiated by data? **Y** 5. Is there coherence between qualitative data sources, collection, analysis, and interpretation? **Y**
Rawlings et al., 2020, Australia	Mixed methods	To clarify the ambiguity about the roles, experiences, skills, and education of death doulas	- In death doulas’ opinions, economizing and registering death doulas would defeat the mission of accessibility inherent to the role - Death doulas believe that healthcare professionals are essential, but that death doulas can work in complement - Death doulas find that they are best acknowledged by healthcare professionals as an extra layer of care rather than replacing anyone’s role	1. Is there an adequate rationale for using a mixed-methods design to address the research question? **Y** 2. Are the different components of the study effectively integrated to answer the research question? **Y** 3. Are the outputs of the integration of qualitative and quantitative components adequately interpreted? **Y** 4. Are divergences and inconsistencies between quantitative and qualitative results adequately addressed? **Y** 5. Do the different components of the study adhere to the quality criteria of each tradition of the methods involved? Y
Tumber, 2020, Canada	Qualitative—phenomenology	- To understand how death doulas fit into the larger framework of death care and death reform - To explore how beliefs concerning gender, death, and social privilege affect the death reform movement - To explore how death doulas may resist or uphold the commodification of their care work	- In some death doulas’ experiences, starting to work with a family as soon as possible is important for building a relationship where death education can occur before death - Death doulas have stated that their experience working between all involved parties is closest to that of a mediator - There are past experiences where death doulas do not “stay in their lane,” which complicates their place in the fabric of palliative and end-of-life care as well as violates healthcare professionals’ trust - Death doulas show discomfort with being paid for their services, and those who describe this payment as a “sliding scale” or work for exchanges but feel in their experience that they wouldn’t be able to turn someone away who could not pay (moral economies)	1. Is the qualitative approach appropriate to answer the research question? **Y** 2. Are the qualitative data collection methods adequate to address the research question? **Y** 3. Are the findings adequately derived from the data? **Y** 4. Is the interpretation of results sufficiently substantiated by data? **Y** 5. Is there coherence between qualitative data sources, collection, analysis, and interpretation? **Y**
Francis, 2021, USA	Qualitative description	To explore the identities of end-of-life doulas through the lenses of gender, class, race, and occupational legitimacy	- Death doula often use intimacy to describe their experiences because it distinguishes it from being “personal” and therefore involving themselves - Death doula cites money transaction as a way to make engagement non-personal - There is a feeling among doula educators from their experience that personalization of care is intrinsic in that you can’t teach someone to just intuit what needs to be done - Some feel as though that a job well done means they no longer need to be a death doula, that is, everyone would be able to go back to this way of dying	1. Is the qualitative approach appropriate to answer the research question? **Y** 2. Are the qualitative data collection methods adequate to address the research question? **Y** 3. Are the findings adequately derived from the data? **Y** 4. Is the interpretation of results sufficiently substantiated by data? **Y** 5. Is there coherence between qualitative data sources, collection, analysis, and interpretation? **Y**
Mallon, 2021, Australia	Qualitative description	- To develop a model for a compassionate community network that expands the current model set forth by Abel et al. - To explore how perceived barriers and challenges of the death doula role could be addressed by a new compassionate community model, the Mallon model	- Some death doulas report that while relationships between themselves and the dying person or family are important, often it’s more about facilitating the creation or support of relationships within he dying person’s network and community - Death doulas’ experience of “slipping in and out” of roles is driven by the dying person’s changing wants and needs - Death doulas find that people’s fear/anxiety around helping a person who is dying can be alleviated through task-driven advice, that is, identifying something for them to do	1. Is the qualitative approach appropriate to answer the research question? **Y** 2. Are the qualitative data collection methods adequate to address the research question? **Y** 3. Are the findings adequately derived from the data? **Y** 4. Is the interpretation of results sufficiently substantiated by data? **Y** 5. Is there coherence between qualitative data sources, collection, analysis, and interpretation? **Y**
Murphy, 2021, UK	Qualitative description	- To describe the roles of end-of-life doulas, including training - To explore how ACP, legacy work, and DNACPR issues are seen by end-of-life doulas - To understand how end-of-life doulas are integrated into the existing network of healthcare spaces at the end of life	- End-of-life doulas center the dying person and their family and then operate as a mediator for all go-betweens during the end-of-life experience - The dying person/family experience peace when an end-of-life doula can facilitate conversation where “nothing is off the table” - Families report information from end-of-life doulas, providing clarity in what would have been overwhelming and confusing - Families report that an end-of-life doula helped provide respite so that they could focus on loving their family member until the end	1. Is the qualitative approach appropriate to answer the research question? **Y** 2. Are the qualitative data collection methods adequate to address the research question? **N** 3. Are the findings adequately derived from the data? **N** 4. Is the interpretation of results sufficiently substantiated by data? **N** 5. Is there coherence between qualitative data sources, collection, analysis, and interpretation? **Y**
Rawlings et al., 2021, Australia	Qualitative description	To gain the perspective of death doulas on their role within both health and social care environments	- Death doula’s experience of advocating for the patient often feels more like translating between multiple parties - Sometimes, death doulas can feel unwelcome when working with healthcare professionals due to misconceptions about what they do - Other times, death doulas feel well-received by healthcare professionals who don’t have the time they would like to be with the dying person at the end of life - Some death doulas feel extremely uncomfortable with asking for money for their services, but it’s a gray area as they do give so much of themselves to those they are engaging in their services - Some death doulas are against regulation as they feel it would be restrictive to the flexible and personalized nature of their role	1. Is the qualitative approach appropriate to answer the research question? **Y** 2. Are the qualitative data collection methods adequate to address the research question? **Y** 3. Are the findings adequately derived from the data? **Y** 4. Is the interpretation of results sufficiently substantiated by data? **Y** 5. Is there coherence between qualitative data sources, collection, analysis, and interpretation? **Y**
Dellinger Page et al., 2022, USA	Mixed-methods approach—sequential explanatory design	- To record what the time an end-of-life doula spent with a dying person/family looked like - To explore the experiences of end-of-life doulas operating within family dynamics and attitudes toward death and dying	- End-of-life doulas feel as though the ability to provide peace and support is the greatest reward of engaging with dying people and their families - End-of-life doulas report that the “intensity” of their emotions is one of the biggest experiential challenges of their role - End-of-life doulas note financial aspects, such as whether they work for a price to be a prominent challenge - Even with the most emotionally complicated families, end-of-life doulas feel, in their experience, that they can help almost anyone find “one shared experience of love” with the dying person, which facilitates acceptance and more of a safer space to discuss death	1. Is there an adequate rationale for using a mixed-methods design to address the research question? **Y** 2. Are the different components of the study effectively integrated to answer the research question? **Y** 3. Are the outputs of the integration of qualitative and quantitative components adequately interpreted? **Y** 4. Are divergences and inconsistencies between quantitative and qualitative results adequately addressed? **Y** 5. Do the different components of the study adhere to the quality criteria of each tradition of the methods involved? **Y**
Ellison, 2022, USA	Narrative piece about the experience of a death doula	- To share the experiences of an end-of-life doula who has worked with dying people who have intellectual disabilities - To explore how end-of-life doulas can better aid those with intellectual disabilities at the end of life	- With the end-of-life doula as facilitator, family members’ “active” role in the dying process allows existing relationships with their loved ones to become more meaningful through and after death - End-of-life doulas who have worked with family members/disabilities have felt they must act more as a navigator between the health and disability sectors	–
Garces-Foley, 2022, USA	Qualitative description	To understand the development of the non-medical end-of-life movement from end-of-life volunteers to professional end-of-life doulas	- End-of-life volunteers speak about “life review” or the concept of experiencing and eliciting the dying person’s story so that they can feel as though it won’t be lost - End-of-life volunteers often experience the need to “fill the gap” or else fill in where care is lacking, either by families who are burdened by care or in healthcare settings - End-of-life vigilers experience honor in witnessing and aiding the transition from life to death	1. Is the qualitative approach appropriate to answer the research question? **Y** 2. Are the qualitative data collection methods adequate to address the research question? **Y** 3. Are the findings adequately derived from the data? **N** 4. Is the interpretation of results sufficiently substantiated by data? **Y** 5. Is there coherence between qualitative data sources, collection, analysis, and interpretation? **Y**
Hahn and Ogle, 2022, USA	Qualitative description	To further elucidate the roles and motivations for end-of-life doulas	- End-of-life doulas speak to experiencing death in their personal life and feeling like their experiences as end-of-life doulas have given them a sense of grounding or control - Through experience, end-of-life doulas have found that personal emotional preparation is required to be able to then center the family/dying person’s emotions above their own - End-of-life doulas speak to the feeling of bringing peace and happiness to a dying person/family as a “gift” or “powerful” - There is a recognized need to be slightly removed from the emotional “quicksand” of the family or dying person, such that you can be fully present as an end-of-life doula	1. Is the qualitative approach appropriate to answer the research question? **Y** 2. Are the qualitative data collection methods adequate to address the research question? **Y** 3. Are the findings adequately derived from the data? **Y** 4. Is the interpretation of results sufficiently substantiated by data? **Y** 5. Is there coherence between qualitative data sources, collection, analysis, and interpretation? **Y**
Chen and Yang, 2023, China	Narrative article	- To describe the experience of introducing hospice narrative doulas to a clinical hospice environment - To demonstrate how hospice narrative doulas can help people and their families at the end of life	- Before the utilization of hospice narrative doulas, patients felt more distressed by loneliness and isolation than fear of death itself - Hospice narrative doulas’ guidance through reflection on life stories can be seen to help dying people and families reinterpret death and its significance to them	–
DeDiego et al., 2023, USA	Mixed-methods approach—triangulation	To understand the roles and experiences of death doulas operating in the USA	- Many death doulas felt called to the role after their own positive experiences with death doulas in their own time of need - Death doulas report a high level of compassion satisfaction—meaning made through supporting others—from their roles at end-of-life - Death doulas report low levels of burnout	1. Is there an adequate rationale for using a mixed-methods design to address the research question? **Y** 2. Are the different components of the study effectively integrated to answer the research question? **Y** 3. Are the outputs of the integration of qualitative and quantitative components adequately interpreted? **Y** 4. Are divergences and inconsistencies between quantitative and qualitative results adequately addressed? **Y** 5. Do the different components of the study adhere to the quality criteria of each tradition of the methods involved? **Y**
Dellinger Page and Husain, 2023, USA	Mixed-methods approach—sequential explanatory design	To explore the different characteristics of end-of-life doulas certified by INELDA	- End-of-life doulas describe their experiences working within hospices as being a “bridge” between non-medical and medical parties - End-of-life doulas reported experiences of feeling as though they were distrusted by medical staff or felt there was a lack of communication with them as a team member - End-of-life doulas are fearful/wary of regulatory doula bodies - End-of-life doulas feel they are most sought out by those who had negative experiences with end-of-life care systems previously	1. Is there an adequate rationale for using a mixed-methods design to address the research question? **Y** 2. Are the different components of the study effectively integrated to answer the research question? **Y** 3. Are the outputs of the integration of qualitative and quantitative components adequately interpreted? **Y** 4. Are divergences and inconsistencies between quantitative and qualitative results adequately addressed? **Y** 5. Do the different components of the study adhere to the quality criteria of each tradition of the methods involved? **Y**
Hahn et al., 2023, USA	Qualitative description	To illuminate the challenges that face end-of-life doulas during end-of-life doula engagement	- End-of-life doulas can find it difficult to create a boundary between being present and getting emotionally involved - End-of-life doulas often grieve both the loss of the dying person and the eventual loss of connection to their families - A lack of knowledge and death literacy is a main facilitator of fear and anxiety for families - End-of-life doulas can have to play mediator between the dying person, family members, healthcare professionals, etc.	1. Is the qualitative approach appropriate to answer the research question? **Y** 2. Are the qualitative data collection methods adequate to address the research question? **Y** 3. Are the findings adequately derived from the data? **Y** 4. Is the interpretation of results sufficiently substantiated by data? **Y** 5. Is there coherence between qualitative data sources, collection, analysis, and interpretation? **Y**
Incorvaia, 2023, USA	Qualitative description	- To describe the scope of practices, trainings, and functions of end-of-life doulas	- With so many doulas hailing from medical backgrounds, there is sometimes an expressed challenge in drawing a clear role boundary - The experience of being “seen and heard” by an objective end-of-life doula gives way to positive emotions - Being an end-of-life doula requires some intuitiveness and emotional maturity to be able to do what is individually right for every person or family engaged with; this can give end-of-life doulas in training anxiety - End-of-life doulas express the need to constantly reevaluate biases to remain objective with everyone they engage with	1. Is the qualitative approach appropriate to answer the research question? **Y** 2. Are the qualitative data collection methods adequate to address the research question? **Y** 3. Are the findings adequately derived from the data? **Y** 4. Is the interpretation of results sufficiently substantiated by data? **Y** 5. Is there coherence between qualitative data sources, collection, analysis, and interpretation? **Y**
Krawczyk et al., 2023, UK	Qualitative description	To summarize the findings, specifically relating to the experience of being a doula, from the first International End of Life Doula Symposium	- Agreement in experience that death literacy increases calm during end-of-life experiences - Doulas’ experience of their role suggests that it is “heart work” and therefore shouldn’t be regulated, while others believe it is expert knowledge and should be paid for	1. Is the qualitative approach appropriate to answer the research question? **Y** 2. Are the qualitative data collection methods adequate to address the research question? **Y** 3. Are the findings adequately derived from the data? **Y** 4. Is the interpretation of results sufficiently substantiated by data? **Y** 5. Is there coherence between qualitative data sources, collection, analysis, and interpretation? **Y**
Rawlings et al., 2023, Australia	Qualitative—phenomenology	- To understand the experiences of family members and patients who used a death doula for a dying loved one from the perspective of bereaved family members - To determine the benefits and challenges of death doula use - To determine cultural shifts toward death and dying after death doula in terms of attitudes toward death and dying	- Death doula engagement allowed family members to feel prepared to deal with their loved one’s death from both an emotional, practical, and spiritual standpoint - Death doula helped the dying person feel empowered through the facilitation of personhood/autonomy - Death doula engagement empowered family members to want to help others who are struggling with the end of life	1. Is the qualitative approach appropriate to answer the research question? **Y** 2. Are the qualitative data collection methods adequate to address the research question? **Y** 3. Are the findings adequately derived from the data? **Y** 4. Is the interpretation of results sufficiently substantiated by data? **Y** 5. Is there coherence between qualitative data sources, collection, analysis, and interpretation? **Y**
De Campos et al., 2024, USA	Quantitative descriptive study—survey	To determine the experience of hospice staff, patients, and end-of-life doula volunteers who participated in the implementation of a volunteer end-of-life doula pilot program within a hospice setting	- Caregivers felt that engaging with an end-of-life doula gave them more information about what to expect when their loved one was dying *and* made them feel more listened to than when working with just hospice staff	1. Is the sampling strategy relevant to address the research question? **Y** 2. Is the sample representative of the target population? **Can’t tell** 3. Are the measurements appropriate? **Can’t tell** 4. Is the risk of nonresponse bias low? **Can’t tell** 5. Is the statistical analysis appropriate to answer the research question? **No**
Donley and Fannin, 2024, USA	Qualitative description	- To explore death doulas’ understanding of spiritual needs at end-of-life and understand how they provide those needs- To understand the experience of death doulas who navigate working with those who have different spiritual or religious beliefs from them	- Many death doulas, after beginning their practice, decide to seek out additional training in diverse alternative care to personalize their engagement - Many death doulas feel compelled to be a guide through the “transition” they are witnessing - Some death doulas experience a struggle to be impartial when it comes to religious differences, while others believe death is equal for all and that relief is driven by being equipped with new knowledge	1. Is the qualitative approach appropriate to answer the research question? **Y** 2. Are the qualitative data collection methods adequate to address the research question? **Y** 3. Are the findings adequately derived from the data? **Y** 4. Is the interpretation of results sufficiently substantiated by data? **Y** 5. Is there coherence between qualitative data sources, collection, analysis, and interpretation? **Y**
Rawlings et al., 2024, Australia	Mixed methods	To understand health care practitioners’ perceptions of what a death doula is and does	- Healthcare professionals who identified as having worked alongside a death doula hold the work of the death doula in either positive regard due to “good effect,” or feel that they were entirely ineffective - There is some worry about the place for a death doula among healthcare professionals and the network of palliative care - Some healthcare professionals feel offended by the offer of a death doula, as they see it as a failure on their part to do their work	1. Is there an adequate rationale for using a mixed-methods design to address the research question? **Y** 2. Are the different components of the study effectively integrated to answer the research question? **Y** 3. Are the outputs of the integration of qualitative and quantitative components adequately interpreted? **Y** 4. Are divergences and inconsistencies between quantitative and qualitative results adequately addressed? **Y** 5. Do the different components of the study adhere to the quality criteria of each tradition of the methods involved? **Y**
Tay Ying, 2024, Singapore	Autobiographical narrative story	To describe the experiences of one death doula working in Singapore	- Death doula describes coming to end-of-life doula work as a “calling” - Death doula work can be slow, as it often becomes just as much about life planning as end-of-life planning	–
Yoong et al., 2024, Singapore	Qualitative description	To elucidate nursing students’ experience of a death “service-learning program” during a 6-month palliative care service rotation	- Death doula student trainees felt unexpected gratitude at being witnesses during the end of life and felt that death doula training eased some of their fears and misconceptions surrounding death and dying - Death doula student trainees recognized that operating as death doulas allowed them to practice person-centered care that they do not always feel they can do as nurses - Recognized need for death doula-like positions to help ease societal taboos around death and dying	1. Is the qualitative approach appropriate to answer the research question? **Y** 2. Are the qualitative data collection methods adequate to address the research question? **Y** 3. Are the findings adequately derived from the data? **Y** 4. Is the interpretation of results sufficiently substantiated by data? **Y** 5. Is there coherence between qualitative data sources, collection, analysis, and interpretation? **Y**
Yoong et al., 2024, Singapore	Mixed-methods approach—sequential explanatory design	- To evaluate the impact of a death doula service-learning experience for nursing students on students’ competencies in palliative care - To understand the experiences of nursing students enrolled in the service-learning death doula program	- Student death doulas showed improved attitudes toward death after completion of training - Service-learning helped students have a more positive regard toward palliative patients - Student death doulas report that service learning was rewarding as they had a positive impact on patients at the end-of-life - Student death doulas report that their experiences demonstrate the need for a death doula to have emotional control when working in this role	1. Is there an adequate rationale for using a mixed-methods design to address the research question? **Y** 2. Are the different components of the study effectively integrated to answer the research question? **Y** 3. Are the outputs of the integration of qualitative and quantitative components adequately interpreted? **Y** 4. Are divergences and inconsistencies between quantitative and qualitative results adequately addressed? **Y** 5. Do the different components of the study adhere to the quality criteria of each tradition of the methods involved? **Y**

PCM, palliative care ministry; UK, United Kingdom; USA, United States.
